# Antioxidant Properties of Cranberry Leaves and Walnut Meal and Their Effect on Nutritional Quality and Oxidative Stability of Broiler Breast Meat

**DOI:** 10.3390/antiox12051084

**Published:** 2023-05-11

**Authors:** Arabela Elena Untea, Iulia Varzaru, Mihaela Saracila, Tatiana Dumitra Panaite, Alexandra Gabriela Oancea, Petru Alexandru Vlaicu, Iulian Alexandru Grosu

**Affiliations:** 1 Food and Feed Quality Laboratory, National Research and Development Institute for Biology and Animal Nutrition, 077015 Balotesti, Ilfov, Romania; iulia.maros@ibna.ro (I.V.); mihaela.saracila@ibna.ro (M.S.); alexandra.oancea@ibna.ro (A.G.O.); alexandru.vlaicu@ibna.ro (P.A.V.); 2 Nutrition Physiology Laboratory, National Research and Development Institute for Biology and Animal Nutrition, 077015 Balotesti, Ilfov, Romania; tatiana.panaite@ibna.ro; 3 Animal Biology Laboratory, National Research and Development Institute for Biology and Animal Nutrition, 077015 Balotesti, Ilfov, Romania; grosu.iulian@ibna.ro

**Keywords:** cranberry leaves, walnut meal, nutrients, broiler, meat, oxidative stability

## Abstract

Dietary sources of bioactive compounds in animal diets, are the natural way to produce animal food products with improved nutritional quality. The present study aimed to test the hypothesis of a synergistic effect of bioactive compounds of cranberry leaf powder and walnut meal on the nutritional quality and antioxidant compounds of broiler meat. An experiment was conducted on 160 COBB 500 broiler chickens, housed in an experimental hall with permanent wood shave litter in boxes of 3 m^2^. The six dietary treatments were based on corn and soybean meal; three experimental groups were fed diets supplemented with cranberry leaves (CLs) with three inclusion rates (0% in the control group and CL 1% and CL 2%); two experimental groups were fed diets supplemented with walnut meal (WM) with two inclusion rates (0% and WM 6%); and two groups were fed diets with a combination of the selected supplements (CL 1% WM 6% and CL 2% WM 6%). The results show that the experimental groups registered higher concentrations of copper and iron compared with the control group. An antagonist effect was noticed on lipophilic compounds, and the lutein and zeaxanthin concentrations presented a dose-dependent increasing effect under CL influence, while vitamin E concentrations decreased in the same manner. The dietary WM positively influenced vitamin E deposits on breast tissue. The dietary supplements did not produce any effect on the primary oxidation products, but the secondary products were influenced, and the maximum effect on the TBARS values were recorded for the dietary combination of CL 1% and WM 6%.

## 1. Introduction

Antioxidants are inhibitors of the oxidation process being integrated into the body’s defence system. Dietary antioxidants can be different vegetal or animal molecular components (natural antioxidants) or synthetic compounds. Nowadays, natural antioxidants are preferred by consumers; they consider that antioxidant-rich foods offer a natural way of protection against free radical occurrence [1]. Natural antioxidants from plants (such as fruits, vegetables, herbs, and spices) are rich in phenolic compounds, liposoluble vitamins, and antioxidant minerals that are valuable free radical inhibitors for humans and animals. According to other studies, different bioactive compounds can act as reducing agents, free radical terminators, metal chelators, and singlet oxygen quenchers in the process of oxidation inhibition [2]. Searching for new feed additives to replace antibiotic growth promoters in farm animal nutrition led the nutritionists to find many alternative natural plants as possible sources of phytochemicals with antimicrobial, antiviral, antiparasitic, antifungal, antioxidant, and anthelminthic properties [3].

Walnut (*Juglans regia*) is a traditional plant, which grows spontaneously almost all over the world. The fruit consumed unprocessed or in food preparations is considered a significant source of bioactive compounds, such as phenolic acids and flavonoids [4]. Walnut oil is a valuable source of polyunsaturated fatty acids and is extracted for its use in food [5]. After pressing and oil extraction, the meal is obtained. Other authors showed that walnut meal is a rich source of biologically active substances (amino acids, polyunsaturated fatty acids, mineral compounds, and polyphenols) [6].

*Vaccinium* species are reported in the scientific literature as a source of phenolic compounds. In human nutrition, they are present as fresh fruits, processed products, and dietary supplements [7]. Other studies have shown that berry leaves are a poor source of anthocyanins compared to fruits, but the phenolic compounds are more abundant [8]. The leaves and fruits have been used in traditional remedies against colds, urinary tract inflammation, diabetes, and ocular dysfunction [9]. The same authors demonstrated that cranberry leaves (*Vaccinium oxycoccus*) are a valuable source of antioxidants, containing significantly more polyphenols than fruit and fruit-based products. In poultry nutrition, cranberry was used mostly as pomace, being a byproduct of the food industry with a minimum economic value. Even though the effects of cranberry pomace on intestinal health [10] and its immunomodulatory effects [11] and the plasma biochemical profile of broilers [12] have been demonstrated, the impact of dietary cranberry leaves in chicken broiler nutrition has not been well documented.

Meat, represented by edible parts of an animal carcass, plays an important role in human evolution due to its chemical composition. Meat is an essential source of high-quality protein, providing all the essential amino acids with high digestibility and is a source of fatty acids and micronutrients [13]. Despite all evident benefits, some studies link a high meat intake with metabolic disorders and heart diseases making nutritionally improved meat a social necessity due to consumer demands [14]. The strategies for improving meat quality follow two main directions: technologies for incorporating bioactive components or nutritional feeding strategies for farm animals. Dietary sources of bioactive compounds in animal diets are the natural way to produce nutrient-enriched animal food products by improving nutrient utilization and antioxidant status and reducing lipid peroxidation in meat production.

Alternative feed resources of bioactive compounds are currently used in broiler nutrition as health-promoting agents, but also to improve the quality of animal products by enriching them in different nutrients and preventing or delaying oxidative damage. A recent study reported that plants, such as basil, thyme, and sage, used in broiler nutrition as phytogenic feed additives led to significant improvements in meat quality [15]. Others showed that the use of byproducts of the food industry, such as grape pomace, tomato peels, or rosehip meal, have positive effects on the oxidative stability of poultry products [16,17]. These studies showed that there is a clear relationship between the quality of animal nutrition and the health safety of the obtained products.

A possible complementary effect of two powerful sources of natural antioxidants was investigated. The study aimed to test the hypothesis of a possible synergistic effect of the bioactive compounds of cranberry leaf powder (a phytogenic feed additive and coproduct derived from the food industry) and walnut meal (a byproduct derived from the food industry) on improving the nutritional profile of broiler meat.

## 2. Materials and Methods

### 2.1. Experimental Design

The experimental study was performed on 240 unsexed, hybrid COBB 500 broiler chickens and lasted 30 days (12–42 days). Legislation regarding animal welfare (Romanian documents 206/2004, 43/11 April 2014, and Directive 2010/63/EU) was the basis for the experimental design used in this study. It was approved by the Ethical Committee of the National Research Development Institute for Biology and Animal Nutrition. One-day-old broiler chicks were purchased from a commercial hatchery and housed in an experimental hall with permanent wood shave litter (10–12 cm thick), simulating the semi-intensive system conditions, in boxes of 3 m^2^ (40 broilers/group/box). They had free access to feed and water. The broilers were weighed individually and assigned to six homogeneous groups based on body weight, following a 2 × 3 factorial arrangement. Microclimate parameters (temperature, humidity, and ventilation) were monitored throughout the experiment. During the first 3 days, the room temperature was maintained at 33 °C and then gradually reduced to 22 °C during the entire experimental period ensuring an average temperature of 26.84 ± 3.45 °C. The light program was 23 h light/1 h darkness.

The six dietary treatments were based on corn and soybean meal; three experimental groups were fed diets supplemented with cranberry leaves (CLs) with three inclusion rates (CL 0%, CL 1%, and CL 2%); two experimental groups were fed diets supplemented with walnut meal (WM) with two inclusion rates (WM 0% and WM 6%); and two groups were fed diets with a combination of the selected supplements (CL 1% WM 6% and CL 2% WM 6%). The group with CL 0% and WM 0% was considered the control group. The broilers had free access to feed and water. Diet formulations are presented in Table 1.

At the end of the experiment (42 days), 6 broilers randomly selected from each treatment were sacrificed by cervical dislocation. Breast meat samples were collected to determine the nutritional quality and the oxidative stability parameters. The samples were stored in the freezer at −80 °C until further analysis.

### 2.2. Chemical Analysis

The proximate composition (crude protein, crude fat, crude fibre, and crude ash) of vegetal materials and meat samples was determined using the methods and equipment described by Untea et al. [18]. Kjeldahl reference method and a semiautomatic Kjeltek auto 1030—FOSS Tecator AB (Höganäs, Sweden) were used for crude protein determinations; continuous extraction in solvent, followed by fat measurement with Soxhlet FOSS Tecator AB (Höganäs, Sweden) was the method used for crude fat concentrations, and gravimetric methods according to Regulation (CE) no. 152/2009 were applied for dry matter content and crude ash.

Flame atomic absorption spectrometry (FAAS) was used to determine trace minerals following the method described by Untea et al. [19] and using a Thermo Electron—SOLAAR M6 Dual Zeeman Comfort (Cambridge, UK). The sample preparation was performed by applying a microwave digestion method manufactured by Bergof (Eningen, Germany).

The lipophilic antioxidant compounds (vitamin E, lutein, and zeaxanthin) were determined using chromatographic methods. The extraction procedures and determination parameters were described by Varzaru et al. [20]. The pretreatment of samples consisted of hydrolyzation with ethanolic potassium hydroxide solution and extraction with petroleum ether. The extracts were washed with distilled water and evaporated under vacuum until dry. The residue was dissolved in ethanol, and the determination was performed using an HPLC system (high-performance liquid chromatography) (Finningan Surveyor Plus, Thermo-Electron Corporation, Waltham, MA, USA) with a HyperSil BDS C18 column.

Spectrophotometric methods (Folin–Ciocalteu and DPPH (2,2-Diphenyl-1-picrylhydrazil)) were used to determine polyphenol content and antioxidant capacity from vegetal materials and meat samples. The extraction details (1 g of dried sample in 10 mL of methanol 80%) and calibration curves were described by Untea et al. [21].

To quantify the oxidative stability of breast samples, the following parameters were considered: primary oxidation products (peroxide value, conjugated dienes, and trienes) and secondary oxidation products (p anisidine value and TBARS (thiobarbituric acid reactive substances)). Furthermore, markers of lipid peroxidation considered the myoglobin fraction (metmyoglobin, deoxymyoglobin, and oxymyoglobin). All the parameters mentioned above were spectrophotometrically determined using a V-530 Jasco manufactured by Japan Servo Co. Ltd. (Tokyo, Japan) spectrophotometer, according to the methods described by Untea et al. [18].

Individual phenolic compounds were identified and quantified using a liquid chromatographic method. The measurements were performed on a Vanquish Core HPLC system equipped with a DAD manufactured by Thermo Fisher Scientific (Bremen, Germany) and BDS HyperSil C18 column (250 × 4 mm, 5 µm particle size) Thermo Fisher Scientific (Bremen, Germany). The binary gradient was prepared from acetic acid (1%) in distilled water (*v*/*v*) (solvent A), methanol (solvent B), and acetonitrile (solvent C) with a flow rate of 0.5 mL/min. The elution program consisted of: 0–15 min: 5% solv B, 5% solv C; 15–20 min: 4% solv B, 15% solv C; 20–25 min: 3% solv B, 25% solv C; 25–40 min: 2% solv B, 38% solv C; 40–50 min: 5% solv B, 5% solv C. The phenolic compounds were identified by overlaying sample peaks with individual reference standards.

Extraction procedure for individual phenolic compound determination followed the next steps: 0.5 g of vegetal powder was extracted by 10 mL of extraction solvent (water/methanol/acetic acid in the ratio of 69:30:1, *v*/*v*/*v*) in screw-cap test tubes, and the extracts were incubated for 60 min at 50 °C in shaking water bath manufactured by Memmert (Schwabach, Germany), followed by centrifugation at 4000 rpm for 15 min [22].

Gallic acid, syringic acid, protocatechuic acid, vanillic acid, ellagic acid, 3-Hydroxybenzoic acid, chlorogenic acid, caffeic acid, ferulic acid, cinnamic acid, methoxycinnamic, catechin, epicatechin, epigallocatechin, quercetin, rutin, and resveratrol were used as standards for polyphenol identification and quantification and were purchased from Sigma-Aldrich (Steinheim, Germany).

### 2.3. Data Analysis

The experimental data were statistically analysed as a 2 × 3 factorial arrangement using two-way ANOVA (General Linear Model procedure) followed by Tukey’s HSD test. The model included the main effects of the cranberry leaves and walnut meal, as well as the interaction between them. XLStat produced by Addinsoft (New York, NY, USA) software was used for statistical interpretation. *p* values below 5% were considered significant. The broilers were weighed at the end of the experiment, with the normality of data being checked by Gruber’s test and the outliers being removed. From each group of animals, six broilers were randomly selected, and each of them become an experimental unit for the meat quality parameter evaluation.

## 3. Results

### 3.1. Chemical Composition of Dietary Supplements

The proximate composition of dietary supplements (Table 2) showed that walnut meal is a valuable source of crude protein and crude fat. Important concentrations of iron were determined in both vegetal materials, with walnut meal presenting also high values of zinc and cranberry leaves being a rich source of manganese.

The concentrations of lipophilic antioxidants of the supplements proved that cranberry leaves are the main source of bioactive compounds from this category (Table 3).

The hydrophilic antioxidants are represented in Table 4 by the phenolic compound profiles. The walnut meal is a vegetal source rich in phenolic acids, particularly ferulic acid (the most abundant phenolic compound in its structure), while CL showed important concentrations of gallic acid and 3-Hydroxybenzoic acid. Cranberry leaves presented higher concentrations of flavonoids, with epicatechin being the main phenolic compound. Rutin is the main flavonol compound both in the WM and CL supplement materials.

### 3.2. Proximate Composition of Breast Tissue

The results regarding the chemical composition of breast meat (Table 5) show a significant effect of the walnut meal presence in the diet, leading to increased fat concentrations. In contrast, the cranberry leaves produced a decreased dose-dependent effect on breast tissue fat concentrations. Overall, the lowest fat concentration was registered for the group fed with 2% CL and 0% WM. No significant differences were recorded on other proximate parameters.

### 3.3. Trace Element Composition of Breast Tissue

The individual inclusion of dietary CL and WM increased the Cu level in the breast meat compared with the control group. The combination of the two supplements also increased the copper level compared with the control group but were significantly decreased when compared with the groups where CL and WM were added individually (Table 6). CL dietary supplements increased Fe deposition in the breast meat, but WM did not influence it. Thus, the increased level of Fe observed in groups supplemented with the combination of CL and WM is due to the influence of CL. Both individually and in combination, WM and Cl decreased Zn storage in the breast meat.

### 3.4. Antioxidant Profile of Breast Meat

Table 7 presents the antioxidant profile of breast tissue as lipophilic bioactive compounds (vitamin E, lutein, and zeaxanthin) and hydrophilic compounds (total polyphenol content). An antagonist effect was noticed between the lipophilic compounds, and under the CL influence, the lutein and zeaxanthin concentrations increased in a dose-dependent manner. The vitamin E concentrations decreased in the same manner, but the presence of WM in the diets led to an increasing effect. The WM inclusion in diets led to decreased polyphenol accumulation on breast tissue levels, and a positive effect of CL was noticed on the antioxidant capacity of the samples.

### 3.5. Oxidative Stability of Breast Meat

The dietary supplements did not produce any effect on the primary oxidation products, but the secondary products were influenced (Table 8). For the maximum positive effect on the inhibition of peroxidation, a synergistic effect of the dietary combination of CL1% and WM 6% was noticed. An inhibitory effect toward the metmyoglobin fraction was noticed in the WM-supplemented groups.

### 3.6. Partial Least Squares-Discriminant Analysis (PLS-DA)

**Figure 1:**
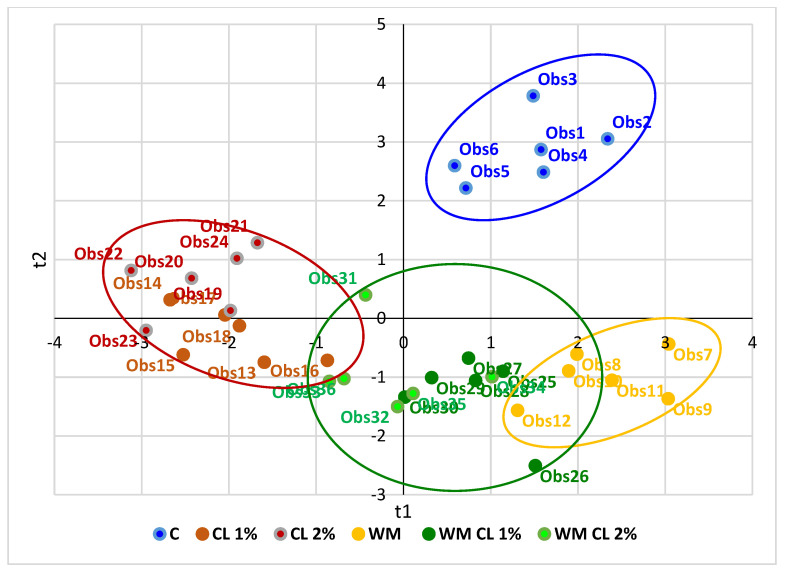
PLS-DA score plot representing the differences between samples belonging to different groups. C—control group; CL—cranberry leaf group; WM—walnut meal group; WMCL—walnut meal plus cranberry leaf group; obs—number of observation.

**Figure 2:**
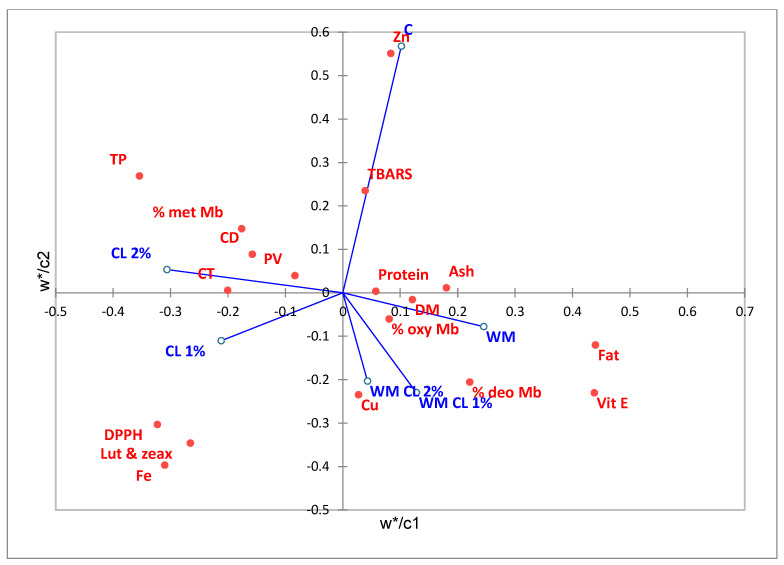
PLS-DA score plot representing the correlations between groups and the chemical markers. C—control group; CL—cranberry leaf group; WM—walnut meal group; WMCL—walnut meal plus cranberry leaf group; Zn—zinc; Fe—iron; Cu—copper; vit E—vitamin E; TP—total polyphenolic content; CD—conjugated diene; CT—conjugated triene; PV—peroxide value; Lut and zea—lutein and zeaxanthin; DM—dry matter; met Mb—metmyoglobin; oxy Mb—oxymyoglobin; deo Mb—deoxymyoglobin. The map overlaps the dependent variables (groups—c vectors) and the explanatory variables (determined parameters—w* vectors) and presents the relationship between the variables.

A multivariate data analysis technique (PLS-DA) was used to establish the correlation pattern between groups and the analytical data. Furthermore, the PLS-DA score plot indicates the similarities and differences between samples belonging to different groups. A far distance between locations in the score plot represented greater differences between groups. The data presented in Figure 1 show that the experimental samples were clustered very well at the bottom of the plot compared to the control, in different quadrants. In the bottom left part of the diagram, the samples belonging to the CL groups were grouped separately and in different quadrants with the WM group. All three groups, which included WM in the diets, were in the same area. The presence of cranberry leaves in the broilers’ diets is strongly associated with antioxidant parameters, such as total polyphenols, DPPH values, and lutein and zeaxanthin concentrations, but also with primary oxidation products, iron concentrations, and metmyoglobin (Figure 2). Walnut meal (single or in mixture) can be related to the fat and fat-soluble vitamin E, copper, and the other two forms of myoglobin. The control group is associated in its quadrant with zinc and TBARS concentrations.

## 4. Discussion

### 4.1. Chemical Composition of Dietary Supplements

Cranberry leaves and walnut meal represent potentially useful feed additives in broiler diets due to their nutritive values and the potential health-promoting effects. The scientific literature is not abundant in data regarding the chemical composition of our studied vegetal material. Considering the growth environment differences, we expected to find slightly different profiles of the chemical composition of plants compared to the data already reported. The proximate composition of cranberry leaves showed that this vegetal material is a poor source of protein or fat. This observation was noticed also by other authors who found that cranberry pomace is a low source of protein [23]. The data presented in our study prove that cranberry leaves are an important source of iron and manganese, results that agree with the mineral profile presented by Karlsons et al. [24] even if the manganese concentrations reported are three times higher than our determination. Oszmianski et al. [9] compared the bioactive content of cranberry fruits, pomace, and leaves from three varieties. They found that the antioxidant potential of leaves is two times higher than fruits and pomace. The DPPH concentrations reported in the leaf samples ranged between 44.4 and 44.6 mM, slightly smaller than our determined values. The antioxidant profiles of cranberry leaves presented higher concentrations of total polyphenols but also of lipophilic antioxidants, which are results confirmed by other authors who found values in the same range [20]. Regarding the walnut meal’s chemical composition, this byproduct is a valuable source of crude protein, crude fat, and minerals, such as iron and zinc. Other authors confirmed that walnut meal is a rich source of protein, accounting for up to 24% of its weight [25]. Vlaicu et al. [26] reported 34.5% crude protein and 12.3% crude fat content in walnut meal dry basis. The crude fat composition contained over 70% polyunsaturated fatty acids, 10% being the omega 3 form [27].

The phenolic compound profiles of the studied vegetal materials consist of five main phenolic groups: hydroxybenzoic acids, hydroxycinnamic acids as phenolic acids, flavonols, flavanols as flavonoids, and stilbenes.

For CL, the most abundant compounds were represented by flavanols, epicatechin being the major compound, followed by hydroxybenzoic acids. The same observation was reported in the scientific literature by other authors, who found that flavonoids are the most relevant group of phenolics in cranberry plants [7,8]. In a recent study, the authors concluded that even if berry fruits are known to present certain health benefits for human consumption, the potential of bioactive compounds from berry byproducts, such as leaves, is not fully investigated regarding bioavailability and the mechanism of action [28].

The walnut meal phenolic profile showed that hydroxycinnamic acids are the main class of compounds, ferulic acid being the major contributor. Walnut leaves and husks were studied, and the authors found that the tannins (gallotannins and ellagitannins) and naphthoquinone derivative juglone are the main active ingredients [29]. Other authors found that the main phenolic acids present in the nuts are ferulic, vanillic, syringic, synaptic, coumaric, caffeic, and protocatechuic, with gallic acid and derivatives being abundant in walnuts [30].

### 4.2. Proximate Composition of Breast Tissue

Diet supplementation with CL, WM, or their combination did not change the proximate composition of breast meat in terms of dry matter, crude protein, or crude ash. The fat concentrations were decreased under CL influence and increased under WM influence, compared with the CL groups. Considering that the fat concentration of WM is eight times higher than CL (Table 2) and the inclusion rate of the two considered feed additives (6% WM vs. 1% or 2% CL), the higher fat deposition in the WM group’s breast meat is an expected result. Using cranberry and blueberry pomaces in broiler diets, Xu et al. [31] reported no effect of dietary berries on the proximate composition of the breast meat. The abdominal fat of broilers was significantly decreased in an experiment that used dietary sumac berries leading to reduced low-density lipoproteins in the blood. The results were reported by [32], who considered that the fat reduction was due to the inhibition of regulatory enzymes involved in cholesterol synthesis or the presence of saponins, which prevent its absorption.

### 4.3. Trace Element Composition of Breast Tissue

It is well recognized that diet is a key factor for chronic diseases; a mixed diet containing meat providing high-quality proteins and minerals could prevent illness [33]. Iron is a trace element involved in the development of the central nervous system, the antioxidant defence system as part of catalase and peroxidase enzymes, and in erythropoiesis, as a part of myoglobin and haemoglobin [34]. The increased level of iron observed in the groups containing the combination of CL and WM was attributed to the presence of CL. Even if WM could be considered a valuable source of minerals, presenting higher concentrations of Cu, Fe, and Zn than CL, the mineral deposition in the breast meat did not respect that pattern. The chemical antagonism of metals, in this case, Fe and Zn, can be noticed in breast meat samples; increased concentrations of iron (all experimental groups compared to the control group) are associated with decreased zinc deposits.

### 4.4. Antioxidant Profile of Breast Meat

The antioxidant profile of CL presented a direct effect on the antioxidant capacity of the breast tissue. No significant effect, compared with the other groups, was noticed regarding the WM presence in the diets. The CL-supplemented groups developed the maximum antioxidant potential, and the combination of the two studied feed additives did not lead to a combined antioxidant effect, even if the WM is also a valuable source of bioactive compounds. The CL-supplemented groups showed increased lutein and zeaxanthin concentrations in a dose-dependent manner without WM influence. Regarding vitamin E concentrations, the same groups registered significantly decreased concentrations in a dose-dependent manner, but a significant antagonist effect was noticed under the WM influence. The combination of the supplements had a positive effect both on vitamin E and lutein and zeaxanthin concentrations, compared with the control group. The antagonist behaviour of vitamin E and xanthophylls was previously noticed by Saracila et al. [35] in a study regarding the effects of chromium picolinate and wooden sorrel dietary supplements on the nutrient quality of broiler meat. The polyphenol concentrations were not influenced by the CL dietary supplements A significant inhibitory effect was noticed when the combination of feed additives was used. Overall, the study on the antioxidant composition of breast meat proved a competing behaviour of selected phytoadditives when they were added in combination and maximum concentrations when they were administrated alone.

### 4.5. Oxidative Stability of Breast Meat

The effects of the supplements added to the diet of broiler chickens on lipid peroxidation of breast meat were seen only in the secondary products of oxidation. Thus, when CL was added to the diet alone, it did not influence lipid peroxidation, but CL (1%) in combination with WM delayed lipid peroxidation, observed by decreasing the TBARS (thiobarbituric acid reactive substances) concentration in breast meat.

Myoglobin, haemoglobin, and cytochrome c are responsible for meat colour. The bright red colour of meat is the result of the oxygenated form of myoglobin (oxymyoglobin). In the absence of oxygen, oxymyoglobin is converted to deoxymyoglobin, which is responsible for the purple-red colour of meat, and by oxidation of iron from the second to the third state, metmyoglobin is formed, producing the brown colour of meat [30]. The improving colour of meat is related to the reduction in metmyoglobin presence. In our study, the presence of walnut meal in the broilers’ diets led to a significant decrease in the metmyoglobin fraction and a positive effect on the deoxymyoglobin fraction, which reflects the presence of iron in the second state of oxidation.

Lipid peroxidation is the main factor affecting the sensory and nutritional quality of meat, and iron is the major catalyst of this process. In muscle tissues, iron can exist in a “free” form (nonheme) or protein-bound (heme) form, and the peroxidation catalysing mechanism was not fully established [36]. The relationship between lipid and myoglobin oxidation was previously explored, and potential mechanisms that could explain the interaction were proposed. Relevant studies considered for many years that metmyoglobin is the heme protein responsible for the initiation of lipid oxidation in muscle tissue. If the red colour of the meat can be maintained, the deterioration process will be delayed [37]. Under physiological conditions, metmyoglobin does not have a pro-oxidative behaviour, but at an acidic pH and mediated by lipid hydroperoxide, metmyoglobin becomes an effective pro-oxidant [38]. Lipid hydroperoxides increase during the conversion of muscle to meat [37], and other studies reported that lipid oxidation preceded oxymyoglobin oxidation [39].

The interrelation between the discussed oxidation processes highlighted the observation that dietary vitamin E delayed both lipid and myoglobin co-oxidation [40]. The antioxidant effect of vitamin E against lipid oxidation is well documented. In contrast, the delaying of oxymyoglobin oxidation was difficult to explain.

The limitations of the study are related to the fact that the feed additives selected are not easily affordable for farm production. Another limitation of the study is the chemical composition of the plants; the study was not focused on possible antinutritional factors present or the bioavailability of the nutrients. Other limitations include the factors affecting the effective dose of phytochemicals including the chemical structures of phytochemical supplements/additives, animal diet, and animal physiological status.

## 5. Conclusions

The results of the present study prove that the inclusion of cranberry leaves and walnut meal in broiler diets represents a nutritional solution rich in bioactive compounds, especially antioxidant constituents (liposoluble antioxidants, phenolic acids, and flavonoids), with a significant improvement effect on the antioxidative properties and on retarding the lipid peroxidation of meat.

## Figures and Tables

**Table 1:** Diet formulations.

Ingredients	Grower Stage (12 to 22 Days)						Finisher Stage (23 to 42 Days)					
	WM 0%			WM 6%			WM 0%			WM 6%		
(%)	CL0%	CL1%	CL2%	CL0%	CL1%	CL2%	CL0%	CL1%	CL2%	CL0%	CL1%	CL2%
Maize	42.00	42.00	42.00	42.00	42.00	42.00	42.00	42.00	42.00	42.00	42.00	42.00
Wheat	18.73	16.77	14.82	15.91	13.96	12.00	20.56	18.6	16.65	17.75	15.79	13.84
SBM	30.53	30.90	31.27	27.37	27.74	28.11	28.10	28.45	28.93	25.03	25.40	25.78
Oil	4.10	4.68	5.25	3.96	4.54	5.12	5.11	5.67	6.26	4.97	5.55	6.12
WM	0	0	0	6.00	6.00	6.00	0	0	0	6.00	6.00	6.00
CL	0	1.00	2.00	0	1.00	2.00	0	1.00	2.00	0	1.00	2.00
Lysine	0.19	0.19	0.18	0.13	0.12	0.12	0.09	0.09	0.08	0.03	0.02	0.02
Methionine	0.24	0.25	0.25	0.30	0.30	0.31	0.20	0.20	0.20	0.25	0.26	0.26
Threonine	0.03	0.03	0.03	0.1	0.10	0.10	0.10	0.10	0.10	0.07	0.07	0.07
CaCO_3_	1.29	1.29	1.29	1.30	1.29	1.29	1.17	1.17	1.17	1.18	1.17	1.17
Ca(H_2_PO_4_)_2_	1.48	1.49	1.50	1.52	1.53	1.54	1.3	1.35	1.32	1.35	1.36	1.37
Salt	0.36	0.36	0.36	0.36	0.36	0.37	0.33	0.33	0.33	0.33	0.34	0.34
Choline	0.05	0.05	0.05	0.05	0.05	0.05	0.04	0.04	0.04	0.04	0.04	0.05
Premix	1.00	1.00	1.00	1.00	1.00	1.00	1.00	1.00	1.00	1.00	1.00	1.00
Total	100	100	100	100	100	100	100	100	100	100	100	100
Chemical theoretical analysis												
ME, Kcal/kg	3086	3086	3086	3086	3086	3086	3167	3167	3167	3167	3167	3167
Crude protein, %	20.00	20.00	20.00	20.00	20.00	20.00	19.00	19.00	19.00	19.00	19.00	19.00
Crude fat, %	5.93	6.51	7.08	6.68	7.25	7.83	6.92	7.49	8.06	7.67	8.24	8.81
Crude fibre, %	3.78	3.94	4.10	4.62	4.78	4.94	3.75	3.87	4.03	4.55	4.71	4.87
Ca (%)	0.84	0.84	0.84	0.84	0.84	0.84	0.76	0.76	0.76	0.76	0.76	0.76
Av. P, %	0.42	0.42	0.42	0.42	0.42	0.42	0.38	0.38	0.38	0.38	0.38	0.38

Premix composition per kg feed: 11.000 IU/kg vitamin A; 2.000 IU/kg vitamin D3; 27 IU/kg vitamin E; 3 mg/kg vitamin K; 2 mg/kg vitamin B1; 4 mg/kg vitamin B2; 14.85 mg/kg pantothenic acid; 27 mg/kg nicotinic acid; 3 mg/kg vitamin B6; 0.04 mg/kg vitamin B7; 1 mg/kg vitamin B9; 0.018 mg/kg vitamin B12; 20 mg/kg vitamin C; 80 mg/kg Mn; 80 mg/kg Fe; 5 mg/kg Cu; 0.60 mg/kg Zn; 0.37 mg/kg Co; 1.52 mg/kg I; 0.18 mg/kg Se. SBM—Soybean meal; CaCO_3_—Calcium carbonate; Ca(H_2_PO_4_)_2_—Monocalcium phosphate; ME—Metabolizable energy; Ca—Calcium; Av. P—Available phosphorus.

**Table 2:** Nutrient composition of dietary phytogenic additives used in experiment (*n* = 3).

Specification	CL	WM
Proximate composition		
Dry matter (%)	93.11 ± 0.72	92.88 ± 0.76
Crude protein (%)	6.63 ± 0.76	29.47 ± 1.03
Crude fat (%)	2.52 ± 0.46	16.24 ± 1.14
Crude fibre (%)	20.15 ± 1.27	18.41 ± 1.56
Crude ash (%)	3.0 ± 0.36	3.88 ± 0.75
Mineral composition		
Copper (mg/kg)	2.182 ± 0.25	19.66 ± 1.18
Iron (mg/kg)	114.6 ± 14.7	225.35 ± 24.2
Zinc (mg/kg)	33.88 ± 3.45	185.21 ± 14.1
Manganese (mg/kg)	448.9 ± 52.1	72.57 ± 5.85

CL—cranberry leaf; WM—walnut meal.

**Table 3:** Lipophilic antioxidants and antioxidant capacity of dietary phytogenic additives used in experiment (*n* = 3).

Specification	CL	WM
Lipophilic Antioxidants and Antioxidant Capacity		
Lutein and zeaxanthin (mg/kg)	348.9 ± 44.5	2.903 ± 0.21
Vitamin E (mg/kg)	205.5 ± 23.4	69.32 ± 4.99
DPPH (mM Trolox equiv)	69.98 ± 3.92	32.77 ± 4.56

CL—cranberry leaf; WM—walnut meal; Lipophilic antioxidants: lutein and zeaxanthin; vitamin E.

**Table 4:** Polyphenolic profile of dietary phytogenic additives used in experiment (*n* = 3).

Specification	Retention Time (min.)	λ (nm)	CL (mg/100 g)	WM (mg/100 g)
Phenolic acids			535.86	971.27
*Hydroxybenzoic acids*			358.28	150.73
Gallic acid (3,4,5-Trihydroxybenzoic acid)	6.29	270	161.6 ± 3.89	70.83 ± 1.84
Vanillic acid (4-Hydroxy-3-methoxybenzoic acid)	23.86	270	15.98 ± 2.81	12.06 ± 1.38
Syringic acid (3,5-Dimethoxy-4-hydroxybenzoic acid)	25.58	270	11.22 ± 1.57	11.93 ± 1.51
3-Hydroxybenzoic acid	25.90	270	149.38 ± 8.75	48.86 ± 2.73
Ellagic acid (Benzoaric acid)	29.84	270	14.54 ± 1.09	n.d.
Protocatechuic acid (3,4-Dihydroxybenzoic acid)	34.11	254	5.53 ± 0.37	7.05 ± 0.25
*Hydroxycinnamic acids*			177.58	820.54
Chlorogenic acid (5-Caffeoylquinic acid)	19.19	280	25.08 ± 1.47	69.34 ± 2.89
Caffeic acid (3,4-Dihydroxycinnamic acid)	24.75	270	16.21 ± 2.27	15.63 ± 1.75
Methoxycinnamic acid	30.45	270	38.07 ± 2.17	37.05 ± 2.16
Ferulic acid (3-Methoxy-4-Hydroxycinnamic acid)	31.62	320	71.88 ± 4.86	698.52 ± 34.3
Cinnamic acid	37.65	270	5.87 ± 0.62	n.d.
Flavonoids			445.16	169.02
*Flavanols*			*415.09*	*154.72*
Epigallocatechin	14.99	270	54.96 ± 3.51	79.79 ± 6.41
Catechin (3,5,7,3′,4′-Pentahydroxyflavane)	18.24	280	52.47 ± 3.87	n.d.
Epicatechin (3,5,7,3′,4′-Pentahydroxyflavane)	26.37	270	307.65 ± 16.1	74.93 ± 3.49
*Flavonols*			*30.07*	*14.30*
Rutin (Quercetin 3-O-rutinoside)	29.28	270	26.39 ± 1.69	14.30 ± 1.63
Quercetin (3,5,7,3′,4′-Pentahydroxyflavone)	35.70	254	3.69 ± 0.49	n.d.
Stilbene			11.38	11.89
Resveratrol (3,5,4′-Trihydroxystilbene)	34.24	254	11.38 ± 1.12	11.89 ± 0.98

CL—cranberry leaf; WM—walnut meal; n.d.—not detected

**Table 5:** The effect of WM and CL dietary supplements on proximate composition of breast meat.

WM	CL	Dry Matter (%)	Protein (%)	Fat (%)	Ash (%)
0	0	22.77	20.21	1.558 ^b^	0.961
	1	22.58	20.31	1.299 ^c^	0.924
	2	21.43	19.25	1.203 ^c^	0.884
6	0	22.69	19.94	1.775 ^a^	0.958
	1	22.73	20.01	1.724 ^a^	0.944
	2	22.79	20.09	1.653 ^a^	0.966
Main effects					
WM					
0%		22.26	19.93	1.353 ^b^	0.923
6%		22.74	20.01	1.717 ^a^	0.956
CL					
0%		22.73	20.08	1.667 ^a^	0.959
1%		22.65	20.16	1.511 ^b^	0.934
2%		22.11	19.67	1.428 ^b^	0.925
***p* value**					
CL		0.599	0.675	0.0001	0.474
WM		0.384	0.849	0.0001	0.167
CL × WM		0.513	0.546	0.061	0.318
SEM					
CL		0.382	0.337	0.030	0.016
WM		0.468	0.412	0.036	0.020
CL × WM		0.662	0.583	0.051	0.029

Means within a column with no common superscript differ (*p* < 0.05). *p*—significance level; SEM—standard error of the mean; CL—cranberry leaf; WM—walnut meal.

**Table 6:** The effect of WM and CL dietary supplements on trace element composition of breast meat.

WM	CL	Cu (mg/kg)	Fe (mg/kg)	Zn (mg/kg)
0	0	24.98 ^d^	22.75 ^d^	37.17 ^a^
	1	26.66 ^b^	30.66 ^ab^	30.54 ^d^
	2	26.62 ^b^	33.40 ^a^	34.24 ^b^
6	0	28.29 ^a^	26.86 ^c^	32.65 ^bc^
	1	25.74 ^c^	30.20 ^abc^	33.17 ^bc^
	2	25.47 ^cd^	29.04 ^bc^	31.48 ^cd^
Main effects				
WM				
0%		26.09 ^b^	28.94	33.98 ^a^
6%		26.50 ^a^	28.70	32.43 ^b^
CL				
0%		26.63 ^a^	24.80 ^b^	34.91 ^a^
1%		26.20 ^b^	30.43 ^a^	31.86 ^b^
2%		26.05 ^b^	31.22 ^a^	32.86 ^b^
***p* value**				
CL		0.006	0.0001	0.0001
WM		0.008	0.732	0.0001
CL × WM		0.0001	0.0001	0.0001
SEM				
CL		0.102	0.492	0.238
WM		0.125	0.603	0.292
CL × WM		0.176	0.852	0.413

Means within a column with no common superscript differ (*p* < 0.05). *p*—significance level; SEM—standard error of the mean; CL—cranberry leaf; WM—walnut meal.

**Table 7:** The effect of WM and CL dietary supplements on antioxidant profile of breast meat.

WM	CL	Total Polyphenols	Vitamin E	Lutein and Zeaxanthin	DPPH
0	0	1.000 ^b^	70.71 ^cd^	0.756 ^d^	1.363 ^c^
	1	1.058 ^b^	59.18 ^de^	1.045 ^bc^	1.836 ^a^
	2	1.301 ^a^	47.44 ^e^	1.260 ^ab^	1.846 ^a^
6	0	0.859 ^bc^	107.31 ^a^	0.845 ^cd^	1.443 ^bc^
	1	0.767 ^c^	91.71 ^ab^	1.021 ^bc^	1.720 ^ab^
	2	0.718 ^c^	86.11 ^bc^	1.398 ^a^	1.630 ^ab^
Main effects					
WM					
0%		1.120 ^a^	59.11 ^b^	1.020	1.682
6%		0.782 ^b^	95.04 ^a^	1.088	1.598
CL					
0%		0.930	89.01 ^a^	0.801 ^c^	1.403 ^b^
1%		0.913	75.45 ^b^	1.033 ^b^	1.778 ^a^
2%		1.010	66.78 ^b^	1.329 ^a^	1.738 ^a^
***p* value**					
CL		0.167	0.0001	0.0001	0.0001
WM		0.0001	0.0001	0.140	0.121
CL × WM		0.001	0.719	0.329	0.082
SEM					
CL		0.030	2.205	0.031	0.037
WM		0.037	2.700	0.038	0.046
CL × WM		0.053	3.819	0.054	0.065

Means within a column with no common superscript differ (*p* < 0.05). *p*—significance level; SEM—standard error of the mean; CL—cranberry leaf; WM—walnut meal.

**Table 8:** The effect of WM and CL dietary supplements on oxidative stability of breast meat.

WM	CL	PV	Diene	Triene	VA	TBARS	metMyo	deoxyMyo	oxyMyo
0	0	0.218	19.28	5.619 ^ab^	26.38	173.2 ^a^	59.58 ^a^	23.64 ^b^	16.88
	1	0.216	19.86	6.140 ^a^	24.66	145.7 ^ab^	61.03 ^a^	23.46 ^b^	15.66
	2	0.222	19.35	5.825 ^ab^	21.19	167.2 ^a^	59.84 ^a^	23.71 ^ab^	16.55
6	0	0.201	15.59	4.214 ^b^	23.26	194.7 ^a^	57.86 ^b^	24.63 ^a^	16.20
	1	0.201	14.85	5.478 ^ab^	19.46	109.7 ^b^	57.37 ^b^	24.95 ^a^	17.84
	2	0.228	21.13	6.066 ^a^	18.89	142.5 ^ab^	58.19 ^ab^	23.95 ^ab^	17.95
Main effects									
WM									
0%		0.219	19.49	5.862	24.08	162.1	60.15 ^a^	23.60 ^b^	16.36
6%		0.210	17.18	5.253	20.54	149.0	58.29 ^b^	24.51 ^a^	17.33
CL									
0%		0.209	17.43	4.917	24.82	183.9 ^a^	59.45	24.13	16.54
1%		0.208	17.35	5.809	22.06	127.7 ^b^	59.20	24.20	16.74
2%		0.225	20.24	5.946	20.04	154.9 ^ab^	59.02	23.83	17.25
***p* value**									
CL		0.497	0.147	0.041	0.116	0.001	0.830	0.449	0.671
WM		0.496	0.092	0.085	0.061	0.229	0.003	0.001	0.153
CL × WM		0.727	0.102	0.161	0.796	0.082	0.072	0.141	0.204
SEM									
CL		0.009	0.938	0.242	1.276	7.524	0.412	0.178	0.466
WM		0.011	1.149	0.296	1.562	9.215	0.505	0.217	0.571
CL × WM		0.016	1.624	0.418	1.987	13.03	0.714	0.307	0.808

Means within a column with no common superscript differ (*p* < 0.05). PV—peroxide value; VA—p anisidine value; metMyo—metmyoglobin; deoxyMyo—deoxymyoglobin; oxyMyo—oxymyoglobin; *p*—significance level; SEM—standard error of the mean; CL—cranberry leaf; WM—walnut meal.

## Data Availability

The data is contained within the article.
